# Photoacoustic Chemical Imaging Sodium Nano-Sensor Utilizing a Solvatochromic Dye Transducer for In Vivo Application

**DOI:** 10.3390/bios13100923

**Published:** 2023-10-11

**Authors:** Jeff Folz, Jacalyn H. Wasserman, Janggun Jo, Xueding Wang, Raoul Kopelman

**Affiliations:** 1 Department of Chemistry, University of Michigan, Ann Arbor, MI 48109, USA; kopelman@umich.edu; 2 Biophysics Program, University of Michigan, Ann Arbor, MI 48109, USA; jhwass@umich.edu; 3 Department of Radiology, University of Michigan, Ann Arbor, MI 48109, USA; janggunj@med.umich.edu (J.J.); xdwang@umich.edu (X.W.)

**Keywords:** ionophore, optode, photoacoustics, PACI, nano-sensor, sodium

## Abstract

Sodium has many vital and diverse roles in the human body, including maintaining the cellular pH, generating action potential, and regulating osmotic pressure. In cancer, sodium dysregulation has been correlated with tumor growth, metastasis, and immune cell inhibition. However, most in vivo sodium measurements are performed via Na^23^ NMR, which is handicapped by slow acquisition times, a low spatial resolution (in mm), and low signal-to-noise ratios. We present here a plasticizer-free, ionophore-based sodium-sensing nanoparticle that utilizes a solvatochromic dye transducer to circumvent the pH cross-sensitivity of most previously reported sodium nano-sensors. We demonstrate that this nano-sensor is non-toxic, boasts a 200 μM detection limit, and is over 1000 times more selective for sodium than potassium. Further, the in vitro photoacoustic calibration curve presented demonstrates the potential of this nano-sensor for performing the in vivo chemical imaging of sodium over the entire physiologically relevant concentration range.

## 1. Introduction

Sodium is one of the most abundant cations in the human body and the cation with the highest concentration in blood [1]. It plays critical and diverse roles in physiology. Sodium is involved in maintaining water distributions in bodily fluid compartments and in regulating osmotic pressure [1,2]. The typical blood concentrations of sodium are around ~140 mM, while cytosolic concentrations are closer to ~5 mM. Through the cotransport of sodium ions, cells are able to maintain cytosolic pH gradients [3]. Sodium also contributes to the proper functioning of the heart and of other muscles, acts as an enzymatic cofactor, and participates in redox reactions [1,2]. As such, sodium is tightly regulated in the body, and disruptions to the typical sodium content can lead to hypertension, heart disease, stroke, and other medical conditions [1].

In cancer, sodium is one of a handful of ions whose physiological regulation is disrupted [3]. The correlation between high sodium concentrations and cancer has been documented in the literature since as early as the 1980s [4], when studies observed elevated salt concentrations in breast cancer tumors [5] and reported correlations between an intracellular influx of sodium and the proliferation of brain cancer [6,7]. In the following decades, the research has further implicated elevated extracellular sodium concentrations in key events in tumor progression, including the Warburg effect, DNA damage, inflammation, and metastasis [3,4,8]. Sodium has also been correlated with an increased expression of the vascular endothelial growth factor (VEGF) via the nuclear factor of activated T-cells (NFAT5), which is a transcription factor involved in regulating intracellular tonicity [4,9]. In turn, VEGF promotes angiogenesis, which is critical in supplying tumors with nutrients [4,10]. Therefore, the literature suggests that elevated sodium in the TME acts as a precursor to cancer-promoting angiogenic processes. Another notable link between sodium and cancer involves isocitrate dehydrogenase (IDH)—a metabolic enzyme involved in the production of NADPH [11]. Sodium measurements have been shown to be able to predict mutations in IDH, which have been demonstrated as an important predictor of glioma treatment response and patient outcomes [11,12,13]. Sodium can also play a role in mediating the immune response in tumors, as elevated sodium levels have increased the prevalence of tumor-associated macrophages, resulting in the exacerbation of cancer progression [14]. Moreover, sodium measurements have been used to differentiate tumor grades [15] and to monitor early responses to therapeutic interventions [16] and are predictive of treatment response in breast cancer [17]. 

Despite the numerous links between sodium concentrations and cancer, the ability to apply this information diagnostically and therapeutically has been limited by the sodium-sensing methods available. The measurement of sodium in vivo is typically carried out via Na^23^ MRI ([11,18], Table S1). While this technique is advantageous due to being non-invasive, the resolution for Na^23^ MRI, in terms of voxel size (volume pixel), is typically limited to around 0.2 mL [19,20,21], while the in-plane resolution is around 1–4 mm [22,23,24,25]. Additionally, Na^23^ has low signal-to-noise ratios, requires expensive, specialized equipment, is inaccessible to patients with metallic implants, and is not conducive to sodium dynamics studies due to long acquisition times [3,24,25]. 

We demonstrate a new method for measuring sodium concentrations using photoacoustic chemical imaging, with the aim of working toward future in vivo chemical imaging [26,27,28]. As pointed out by the method’s pioneer, through combining the merits of the use of both light and sound, PA imaging has the unique capability to present detailed structures and functions in optically scattering biological tissues at unprecedented depths [29]. Photoacoustic chemical imaging (PACI) has been employed to measure other alkaline metals, such as lithium and potassium [26,27,28]. We use a similar approach in which we employ a sodium-sensitive nanoparticle as a contrast agent.

**Figure 1:**
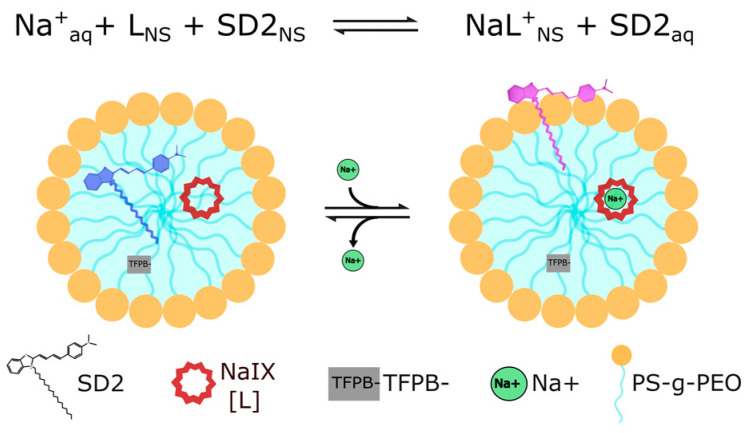
A scheme demonstrating the sensing mechanism of an SDNaNP. The solvatochromic dye (SD2), ionophore (NaIX, L), and counterion (TFPB-) occupy the hydrophobic core of the nano-sensor in the absence of sodium. When sodium from the solution chelates the ionophore, the positively charged head of SD2 is pushed from the interior out to the nano-sensor’s surface, which induces a change in the dye’s spectral absorption. It should be noted that, while depicted as phospholipid-like, the nanocarrier (PS-g-PEO) is a bottlebrush co-polymer.

In this work, we chose a solvatochromic dye as our optical transducer to mitigate pH cross-sensitivity [30,31,32,33,34,35,36]. By employing a bottlebrush co-polymer as the nanocarrier, we can generate a plasticizer-free, ionophore-based nano-sensor [36,37]. The resulting nano-sensor is highly sensitive and selective to Na^+^, is non-toxic, and makes an excellent candidate nanoparticle for in vivo sodium imaging. Figure 1 shows the operating principle. The dye consists of two components: a positively charged dye head whose optical properties are sensitive to the environment and a long fatty tail that keeps the dye anchored in the nanoparticle matrix. As the aqueous sodium concentration increases, sodium is chelated from the solution and incorporated into the nano-sensor’s hydrophobic interior. Subsequently, the positively charged head of the solvatochromic dye is pushed from the interior out to the nano-sensor’s surface, which induces a change in the dye’s spectral absorption. The dye remains anchored in the nano-sensor matrix as a result of its fatty chain, without which the dye would be quickly leached into the solution.

## 2. Results

**Figure 2:**
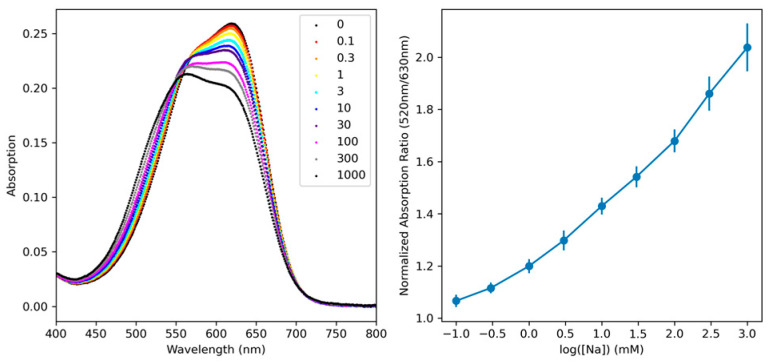
(**Left**): the raw UV-Vis absorption spectrum for the SDNaNPs at different sodium concentrations (in mM). (**Right**): the normalized, ratiometric calibration curve showing the nano-sensor’s response to changes in sodium concentration. Note that the sodium bioconcentration rarely exceeds 200 mM.

The obtained nano-sensors were approximately 45 nm in diameter, which is comparable to other ionophore-based nano-sensors (Figure S1). Due to the polyethylene glycol grafted to the polystyrene backbone of the polymer, the sensors had a zeta potential of −26.4 mV, indicating a high particle stability and a reduced tendency to aggregate (Figure S2). Figure 2 shows how the above changes in spectral absorption can be used for sodium sensing. The left-hand side of Figure 2 shows the raw absorption spectra for the SDNaNP. When the dye resides in the nanoparticle’s hydrophobic interior, its peak absorption is near 630 nm. As the sodium is chelated and the dye is pushed out to the particle’s surface, the absorbance intensity decreases from 630 nm, and there is a blue shift in its absorbance spectrum. As shown in Figure 2, there is an isosbestic point located near 550 nm. As ratiometric sensors are preferred due to their independence from sensor concentration, we wanted to use a secondary wavelength as a reference. We determine that the absorption ratio between 630 nm and 520 nm yields the greatest sensitivity to sodium concentrations. We then normalize this ratio to the ratio observed in a sodium-free solution. By plotting the normalized ratio of the 630 nm absorption peak to that of the 520 nm reference peak, we obtain a calibration curve that can be used to determine unknown sodium concentrations. Figure 2′s right-hand side shows such a calibration, with error bars representing the standard deviation of three separate batches of SDNaNPs. We observe a high degree of sensitivity in the biologically relevant sodium concentration range (~1–150 mM). 

**Figure 3:**
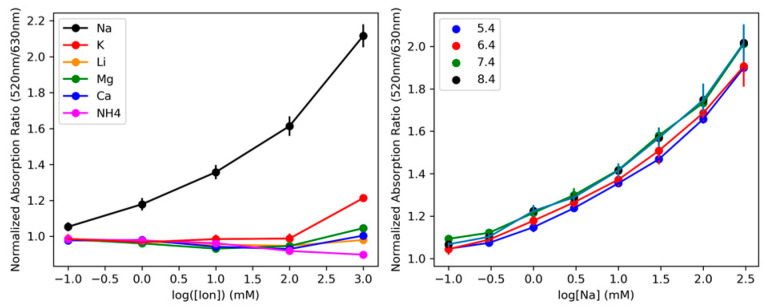
(**Left**): A ‘selectivity’ calibration curve for simple cations that are likely to compete with sodium for binding. Only potassium shows any signal response, and then only at concentrations well above most physiological values. (**Right**): calibrations showing the pH insensitivity of the SDNaNP. The error bars for both calibrations are assumed to be comparable to those in Figure 2. The pH values are indicated by the legend and cover the range of physiologically relevant values.

A critical aspect of any sensor is its selectivity regarding interfering analytes. Figure 3 demonstrates the SDNaNP’s insensitivity to analytes other than sodium. On the left, we see a calibration of the SDNaNP for a variety of simple cations. With the exception of potassium, there is essentially no signal change from the SDNaNP, meaning that the nano-sensor is unaffected by these ions. As potassium is chemically very similar to sodium, it is not unexpected that some response in the SDNaNP is observed at the highest potassium concentrations (1000 mM). Figure S3 shows a calibration of the sensor in different background concentrations of potassium, and the calibration is largely unaffected by physiologically relevant potassium concentrations, where the highest potassium concentrations in the cytosol are ~150 mM. The right-hand side of Figure 3 shows four separate calibrations curves for the SDNaNP, where each was taken in the same buffer titrated to different pH levels. In the biologically relevant range from pH 5.4 to 8.4, we observe no deviation in the SDNaNP calibration, suggesting that common disruptions in the tumor microenvironment’s pH will not affect the accuracy of the sodium measurements taken there. 

**Figure 4:**
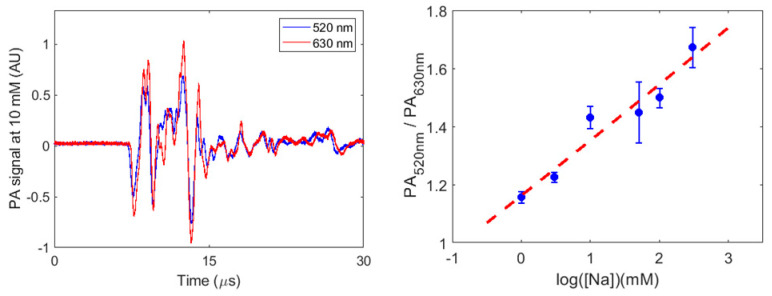
(**Left**): PA signals of the SDNaNPs at 10 mM with 520 nm and 630 nm wavelengths of light excitation. (**Right**): the photoacoustic calibration curve of SDNaNP, using the normalized ratio of PA signals, at 520 nm over that at 630 nm, for 1, 3, 10, 50, 100, and 300 mM of Na^+^. The red dashed line is the line of best fit.

The PA signals from the SDNaNPs at different concentrations of Na^+^ were collected. The ratios of the PA signal intensity, with 520 nm and 630 nm wavelength excitations, were obtained. Figure 4 (left) shows the exemplary PA signals from the SDNaNP at 10 mM of Na^+^ with 520 nm and 630 nm excitation wavelengths. As the absorption of SDNaNP in Figure 2 shows (left), the variation in the PA signal intensity at 630 nm was larger than at the 520 nm wavelength. Figure 4 (right) shows the normalized ratio between the intensities at the two excitations, with 520 nm and 630 nm, of SDNaNPs when mixed with different concentrations (1, 3, 10, 50, 100, and 300 mM) of Na^+^ solutions. The red dotted line indicates the fitting line of the ratio results. The PA ratio shows a logarithmic relationship with the sodium concentration. 

## 3. Discussion

We have presented here a sodium-responsive nano-sensor that utilizes a solvatochromic dye transducer with a phase-transition mechanism for ratiometric sodium measurements. Because sodium is an important biological metabolite with varied roles in physiology, sodium sensing has diverse applications, from water quality testing to nutritional analysis to clinical diagnostics [1,38]. While the SDNaNP can function in many of these roles, our primary aim was to address the current gap in the literature regarding sodium nano-sensors suited to in vivo applications ([3,38], Table S2). Compared to Na^23^ MRI, which is the current standard for in vivo sodium measurements, sodium nano-sensors achieve a higher resolution and a higher signal-to-noise ratio and are less expensive ([3,38], Table S1). However, most of the sodium nano-sensors reported in the literature historically operate on an H^+^/Na^+^ ion-exchange principle that renders them sensitive to pH [38,39,40,41,42,43,44,45]. While variations in blood pH are minimal and can be performed with a pH-sensitive dye given proper controls, such measurements in the acidic tumor microenvironment cannot be reliably performed without the simultaneous measurement of the tumor pH. With our approach, a single photoacoustic probe could reliably measure the sodium concentration in the tumor microenvironment (TME) because the utilization of a solvatochromic dye removes the pH dependency of the signal response mechanism [32]. 

While a previously reported nano-sensor utilizing a Biginelli ligand presented an alternative approach to avoiding pH cross-sensitivity while achieving an impressive detection limit of 22 nM Na^+^, the dynamic range was relatively limited and well below the biologically relevant concentration of sodium [46]. Instead, one may use dye that is directly sensitive to sodium ions [47]. However, when such dyes are commercially available, they tend to be strongly fluorescent at the blue–green end of the visible spectrum, inhibiting their in vivo use due to both their absorption and scattering effects. By employing a solvatochromic dye, we present a nano-sensor with a ratiometric absorbance at the redder portion of the spectrum and demonstrate it to be unaffected by physiologically relevant changes in pH.

The SDNaNP has been presented here in the context of the absorption readout mode. However, it should be noted that the dye, SD2, has a significant quantum yield and has been utilized in the context of a fluorescence reporter. Nevertheless, the emission maximum is in the visible spectrum and is ill-suited to in vivo fluorescence imaging. A future direction for our research will be designing near-infrared solvatochromic dyes to better enable in vivo measurements in all readouts. Moreover, in addition to the absorption readout mode of SDNaNP, we demonstrate its capability of acting as a photoacoustic chemical contrast agent, which would broaden its in vivo penetration limits [29,48]. Further, the ionophore-based contrast agent is able to function without the presence of any plasticizer, thus eliminating a potentially toxic additive. Indeed, the results of our cell viability assay (Figure S4) demonstrate that the SDNaNP is biocompatible. 

One disadvantage of utilizing a positively charged solvatochromic dye is interference from anions. It has been demonstrated that negatively charged anions are able to interact with the positively charged head groups of the dyes and effectively ‘pull’ them from the hydrophobic interior of the nano-sensor toward the surface, which induces an optical response in the sensor identical to that of the target analyte [49]. This interference by anions follows the trend of the Hofmeister series. For in vivo applications, these interactions can be ignored as the strongest effects come from perchlorate, thiocyanate, and iodide, which each have blood concentrations far below the observed interfering effect. It should also be noted that the well-known formation of a protein corona on nanoparticles in the blood may also have effects on the sensor calibration, which will need to be interrogated. A second possible disadvantage of utilizing solvatochromic dyes is the relatively slow response times of these sensors in plasticizer-free systems [37]. While the SDNaNP sensor response time (~3 s, Figure S5) is certainly too slow to visualize the dynamics of action potentials, it is faster than the photoacoustic image acquisition time and among the faster response times of previously reported sensors. However, the seconds time scale is still too slow for probing many dynamic, interesting, and relevant physiological phenomenon. 

In conclusion, we have presented here a non-toxic, highly selective nano-sensor for sodium ions. The utilization of a solvatochromic dye negates the pH dependence commonly found in other formulations of ionophore-based nano-sensors. We demonstrated that this nano-sensor is able to function as a photoacoustic contrast agent that will enable the real-time collection of images that quantitatively map sodium concentrations in the tumor microenvironment (TME). We believe that this nano-sensor is a critical addition to a growing set of nano-sensors enabling photoacoustic chemical imaging.

## 4. Methods

Materials. Unless otherwise indicated, all chemicals were purchased from Sigma-Aldrich (St. Louis, MO, USA). When available, the purity and manufacturer’s serial number are provided in parenthesis. 

Synthesis of Solvatochromic Dye 2. Synthesis of solvatochromic dye 2 (SD2, Figure S6) was accomplished via previously reported methods [28,32]. Briefly, an alkylation reaction was performed on 1.5 g 2-methylbenzothiazole (99%, 112143-25G) using 3.8 g of the alkylating agent 1-iodooctadecane (95%, 251984-25G) dissolved and refluxed in acetonitrile for 24 h. The solution was cooled and allowed to solidify. The crude product was precipitated in diethyl ether, collected, and washed several times in diethyl ether. A total of 265 mg of this product, along with 122 mg (dimethylamino)cinnamaldehyde (D4506-5G), was then dissolved in acetic anhydride and refluxed for 20 min in a Knoevenagel reaction. The reaction solution was then poured into a warm solution of 10 mM sodium iodide (in Millipore water). The dark purple precipitate was washed several times with water, dried, and collected. Figure S7 provides that structure’s H^1^-NMR spectrum.

Preparation and Characterization of SDNaNP Nano-sensor. Solvent displacement was employed to prepare one batch of the nano-sensor. The following methanol stock solutions were prepared: 1 mg/mL SD2 (synthesized in lab), 1 mg/ml sodium tetrakis[3,5-bis(trifluoromethyl)phenyl]borate (NaTFPB) (692360-1G), 10 mg/mL polystyrene-graft-poly(ethylene oxide) (PS-g-PEO, purchased from Polymer Source Inc., Dorval, QC, Canada) (P15020A-SEOComb), and 1 mg/mL mg Sodium Ionopre X (NaIX) (Selectophore^TM^ 71747-50MG). From these methanol solutions, 0.2 mg SD2, 0.9 mg NaTFPB, 5 mg PS-g-PEO, and 1.07 mg NaIX were combined. Then, 1 mL of the resulting cocktail solution mixture was injected into 40 mL of Millipore water, while stirring was carried out under compressed air at upwards of 700 rpm for at least 30 min. The nano-sensor solution was concentrated via centrifuge filtration to 1.5 mL. UV–Vis spectroscopy measurements were taken in 10 mM 3-(N-morpholino)propanesulfonic acid (MOPS), 5 mM KCl, 2.5 mM CaCl_2_, and 1 mM MgCl_2_ at pH 7.4 using a Shimadzu 2600 UV–Vis Spectrophotometer. This buffer was designed to simulate the pH and cation concentrations of extracellular fluid and was also administered in the MTT assay (Figure S4). Of note, the presence of these additional ions was determined not to interfere with the nano-sensor’s measurements via ion selectivity experiments, as described below.

For the cation selectivity experiments, the nano-sensor was prepared as outlined above, and its absorbance was measured in a pH 7.4 MOPS-buffered solution containing the desired concentrations of either NaCl, KCl, CaCl_2_, MgCl_2_, LiCl, or (NH_4_)_2_SO_4_^−^, respectively. The pH sensitivity experiments were performed in solutions containing 10 mM MOPS, 5 mM KCl, 2.5 mM CaCl2, and 1 mM MgCl_2_ titrated to the respective pH with NH_4_OH.

For all the UV–Vis response calibrations, the absorption ratio was calculated via dividing the absorbance at 520 nm by that at 630 nm. The normalized ratiometric absorbance was then calculated via dividing the absorbance ratio at the respective cation concentration by the absorbance ration in the absence of that analyte. Analyte concentrations, unless otherwise indicated, ranged from 0 to 1000 mM, to encompass and far exceed the concentrations expected to be encountered in vivo. 

Toxicity Assay. MTT toxicity assays were conducted on a 96-well plate containing 5000 Hela (ATCC) cells in 0.1 mL of media (Dulbecco’s Modified Eagle Medium supplemented with 10% fetal bovine serum and 1% penicillin/streptomycin). Treatment groups consisted of 8 trial wells, each receiving 11 µL of the corresponding treatment solution: Millipore water (control group), 10 mg/mL nano-sensor solution (resulting in a final incubation concentration of 1 mg/mL NP), 1 mg/mL nano-sensor solution (resulting in a final incubation concentration 0.1 mg/mL NP), 0.5 mg/mL nano-sensor solution (0.05 mg/mL NP treatment), MOPS-buffered saline solution (buffer control), 7 mg/mL PS-g-PEO solution (PS-g-PEO treatment), 0.28 mg/mL SD2 solution (SD2 treatment), or a solution of water and evaporated methanol (methanol control treatment). For the nano-sensor solutions, the SDNaNP nano-sensor was synthesized as described above and diluted in Millipore water to the desired concentrations. The PS-g-PEO and SD2 solutions were prepared via pipetting methanol stock solutions into Millipore water, before compressed air was used to evaporate off the methanol. The concentration of free PS-g-PEO and free SD2 equaled the concentration of these components in the 10 mg/mL nanoparticle solution (1 mg/mL NP treatment). As a metric for the toxicity of any lingering methanol, the methanol control solution was prepared via pipetting an equivalent volume of pure methanol into Millipore water and letting it sit under compressed air for the same amount of time (roughly 30 min).

Following a 24 h incubation, 3-(4,5-dimethylthiazol-2-yl)-2,5-diphenyltetrazolium bromide) (MTT) was added, and the cells were incubated for another 4 h. The MTT crystals were then solubilized with DMSO, and UV–VIS measurements were taken using a plate reader. The average absorbance across the 8 trial wells was calculated and compared to the control group to obtain the cell viability data.

Nano-sensor characterization. Dynamic light scattering (DLS) was performed using a DynoPro NanoStar on 100 µL samples of 0.5 mg/mL SDNaNP at 0–1000 mM Na^+^ in Millipore water. For each sample, 10 DLS acquisitions were performed and averaged. The zeta potential was measured using a Malvern Zetasizer Nano ZSP at a particle concentration of 1 mg/mL. Kinetic measurements were performed at the same particle concentration with 1 M NaCl injections in MOPS-buffered saline on a stopped-flow instrument in absorption readout mode (Tgk Scientific SF-61DX2 KinetAsyst).

Photoacoustic signal collection setup. The synthetized SDNaNP was placed in an optically clear polyvinyl chloride tube (ID 1/16 inch and OD 1/8 inch) and exposed to 520 nm and 630 nm wavelengths of laser light (Nd:YAG, Surelite, Continuum). An optical parametric oscillator (SLOPO Plus, Continuum), pumped with the second harmonic of a pulsed neodymium-doped aluminum garnet laser was used. The PA signal was detected using a 2.25 MHz ultrasonic transducer (V323, Panametrics) and amplified via an amplifier (5072PR, Olympus). The PA signal, digitalized using an oscilloscope (TDS540, Tektronix), was collected (averaged over 50 pulses). The ratios between the signal intensity at 520 nm and 630 nm were used to generate the calibration curve. The normalized ratio shows the ratio of the PA signal intensity between 520 nm and 630 nm at different concentrations, divided by the ratio of SDNaNP at 0 mM (Figure 4).

## 5. Associated Content

Supporting information: Dye characterization, including structure and H^1^-NMR; Characterization of nanoparticles’ size (radius), zeta potential, toxicity, selectivity, and response time (PDF).

## Data Availability

Data is available in an online repository as part of University of Michigan’s DeepBlue Data Depositories, and can be found under deposit ID: rf55z836q.

## Supplementary Materials

The following supporting information can be downloaded at: https://www.mdpi.com/article/10.3390/bios13100923/s1, Figure S1. (A) Average nanosensor size by Na^+^ concentration, as determined by DLS analysis. Bar height represents the average radius across 10 acquisitions, and error bars represent one standard deviation in each direction. Size distribution of unfiltered (B) and filtered (C) nanosensor solution at 0 mM Na^+^, as determined by DLS analysis. The mean unfiltered radius was 35.4 nm with a standard deviation of 4.5 nm. The mean filtered radius was 23.4 nm with a standard deviation of 1.5 nm. Figure S2. A distribution of zeta potentials in a sample of SDNaNPs. Measurements were taken in Millipore water. The average zeta potential of the SDNaNPs is −26.4 ± 3.5 mV, which indicates that the nanosensors are quite stable and reluctant to aggregate. Figure S3. Four calibrations of the SDNaNP with different concentrations of potassium background. From 0 to 100 mM, the presence of potassium has minimal effect on the calibration of the SDNaNP. Potassium concetrations are indicated by the legend and are reported in millimolar units (mM). Figure S4. Results from an MTT assay that probed the toxicity of the nanosensor. No toxicity was observed from the SDNaNP, even at its highest working concentration. Figure S5. Five stopped-flow runs evaluating the response time of the SDNaNPs in absorption mode. The majority of the SDNaNP response happens quickly, with 90% of the total response (t90) occurring with 3.0 ± 0.3 s and t95 being 5.5 ± 0.3 s. Figure S6. Chemical structure of the dye, SD2. Note that the counter anion, iodide, is not drawn here. Figure S7. An H1-NMR spectrum for the synthesized dye, SD2. The NMR spectrum was collected in deuterated chloroform. Table S1. Comparison of the advantages and disadvantages of prominent techniques for in vivo sodium sensing. Table S2. Comparison of SDNaNP to a selection of other prominent sodium nano-sensors reported in the literature. Sensitivity is provided in terms of the detection limit and the dynamic range. Selectivity is given by the selectivity coefficients, a comparison to the potassium concentration required for an equivalent response as to sodium. Fluorescence wavelengths represent the emission wavelengths. References [50,51,52,53,54,55] are only mentioned in the Supplementary Material.
