# Supplementary material for: Photoacoustic Chemical Imaging Sodium Nano-Sensor Utilizing a Solvatochromic Dye Transducer for In Vivo Application
Source: Biosensors (Basel). 2023 Oct 11;13(10):923. doi: 10.3390/bios13100923 (PMC10605089; doi:10.3390/bios13100923)
Supplement: Supplementary file 1 [file biosensors-13-00923-s001.zip › biosensors-2600081-supplementary.pdf]

Photoacoustic Chemical Imaging Sodium Nano-Sensor Utilizing a Solvatochromic Dye Transducer for In Vivo Application

Jeff Folz, Jacalyn H. Wasserman, Janggun Jo, Xueding Wang, Raoul Kopelman

Nanosensor Characterization. Size characterization was performed using Dynamic Light Scattering (DLS). The average SD NaNP radius across the six samples was 47.2 nm. Measurements for all samples (0 mM - 1000 mM) were within error of one another, with no significant, consistent trend between size and sodium concentration observed. Therefore, it can be concluded that the particle size is stable across sodium concentrations of 0 - 1000 mM. While a multimodal distribution was observed at each measured concentration, syringe filtration, which was subsequently performed on the 0 mM sample, was successful at obtaining a unimodal size distribution. Additionally, the removal of the large particles via the filtering process decreased the average particle size to just 23.42 nm in radius.

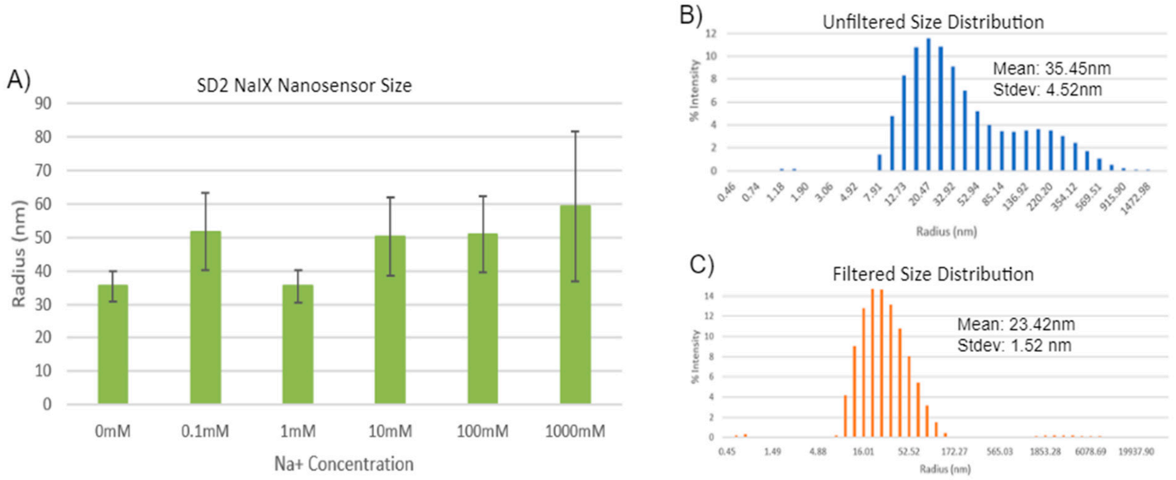

Figure S1. (A) Average nanosensor size by Na+ concentration, as determined by DLS analysis. Bar height represents the average radius across 10 acquisitions, and error bars represent one standard deviation in each direction. Size distribution of unfiltered (B) and filtered (C) nanosensor solution at 0 mM Na+, as determined by DLS analysis. The mean unfiltered radius was 35.4 nm with a standard deviation of 4.5 nm. The mean filtered radius was 23.4 nm with a standard deviation of 1.5 nm.

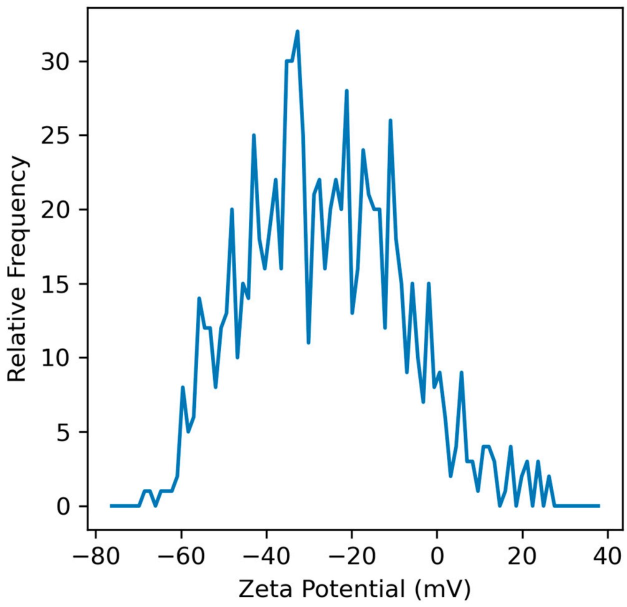

Figure S2. A distribution of zeta potentials in a sample of SDNaNPs. Measurements were taken in Millipore water. The average zeta potential of the SDNaNPs is -26.4 ± 3.5 mV, which indicates that the nanosensors are quite stable and reluctant to aggregate.

Selectivity and Sensitivity. To evaluate and confirm the selectivity of the nanosensor, four separate characterizations were made in MOPS buffered saline with relevant calcium and magnesium backgrounds (see: methods) in the presence of different background concentrations of potassium.

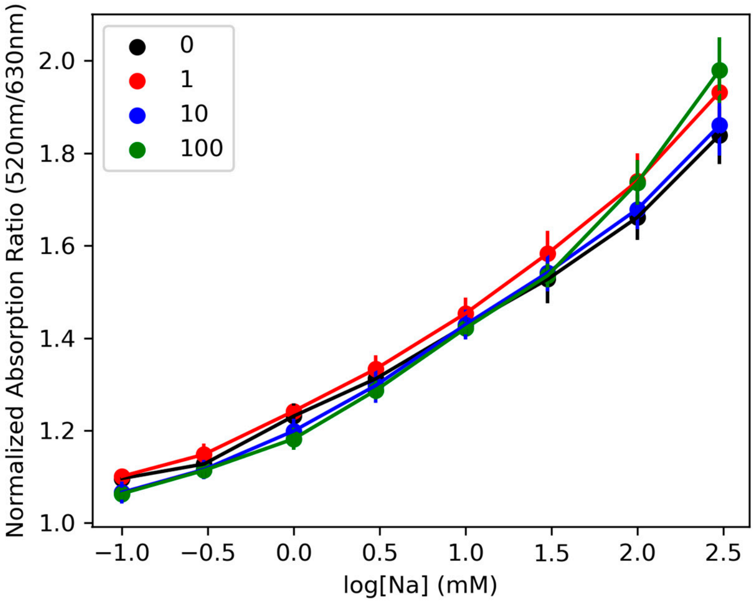

Figure S3. Four calibrations of the SDNaNP with different concentrations of potassium background. From 0 to 100mM, the presence of potassium has minimal effect on the calibration of the SDNaNP. Potassium concetrations are indicated by the legend and are reported in millimolar units (mM).

Through the use of the separate solutions method and the data from figure 3 [38,50], we were able to characterize the selectivity of our sensor for sodium over potassium (k_{NaK}) was found to be -3.2 ± 0.2.

To determine the detection limit of the SDNaNP, we compared, in triplicate, the normalized absorption ratio from a dilute sodium sample (100 pM; assumed to be orders of magnitude below the SDNaNP detection limit) to solutions containing higher amounts of sodium. We then compared the mean and standard deviation of the measurements taken on the dilute sample to the test sample using the student's t-test. We selected as our detection limit the smallest sodium sample whose measurements were statistically different from the dilute samples' at 95% confidence. Using this approach, the detection limit of the SDNaNP was found to be 200 μM.

*Toxicity Characterization***.** We used an MTT cell viability assay to assess any toxic effects induced by the presence of the SDNaNP. 5,000 HeLA cells were incubated with the SDNaNP or its components for 24 hours. Following the 24h incubation, the MTT agent was introduced, and incubated 4 hours before a solubilization agent was added. The results of the toxicity assay can be seen in Figure S3. We observe very little toxicity for the SDNaNP, even at its highest incubation concentration of 1 mg/mL (the estimated working concentration *in vivo*). Interestingly, while the SDNaNP is non-toxic, we observe some toxicity induced by both the bottle brush polymer and the solvatochromic dye. We attribute the reduced toxicity in the nanoparticle formulation of these agents as a reduction in bioavailability, meaning that the dye and polymer remain largely sequestered in the nanoparticle, rather than as free-floating constituents.

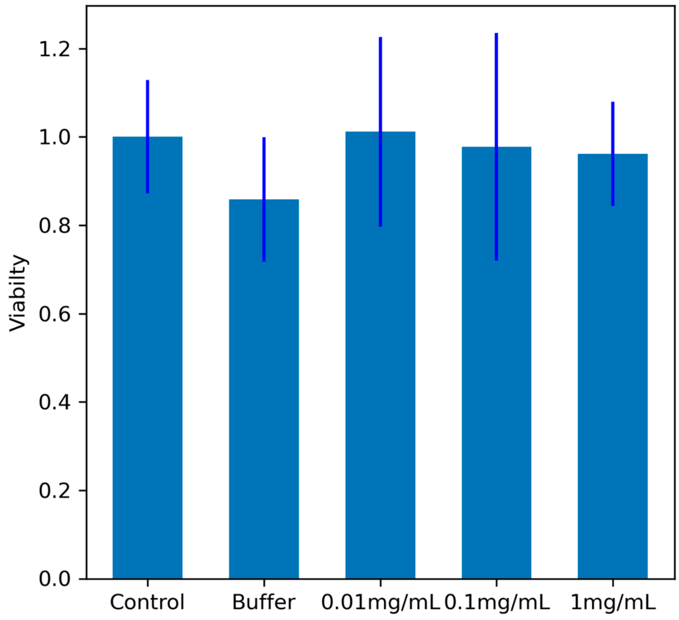

Figure S4. Results from an MTT assay that probed the toxicity of the nanosensor. No toxicity was observed from the SDNaNP, even at its highest working concentration.

Kinetics. Response time was measured using a stopped-flow instrument in absorption readout mode. Five separate injections of 1M NaCl were used to evaluate the sensor response time.

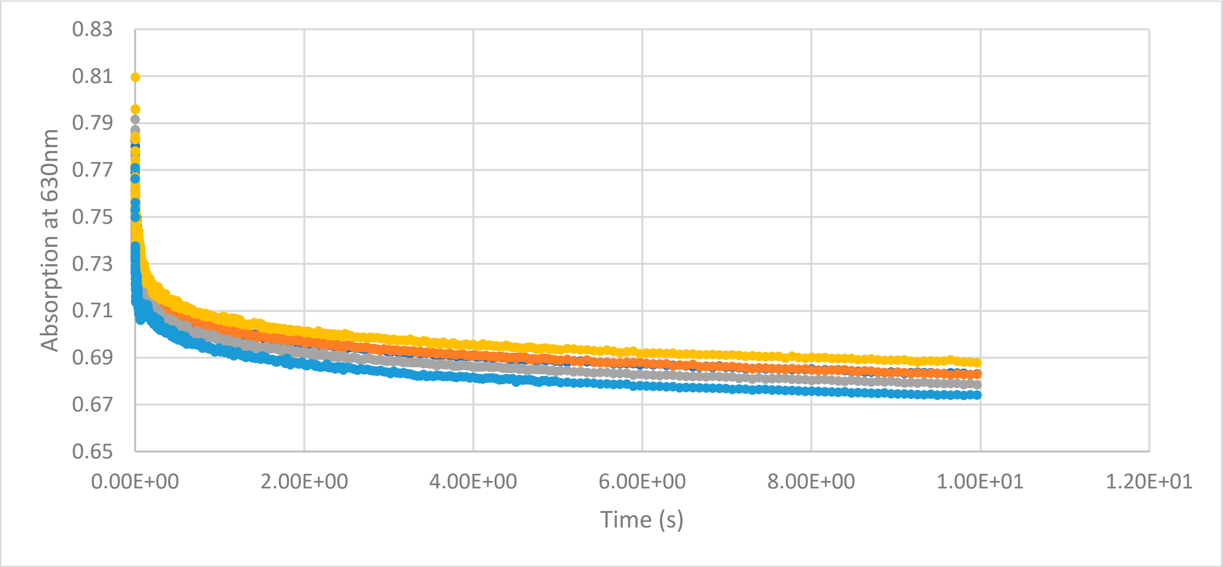

Figure S5. Five stopped-flow runs evaluating the response time of the SDNaNPs in absorption mode. The majority of the SDNaNP response happens quickly, with 90% of the total response (t_{90}) occurring with 3.0 ± 0.3 s and t_{95} being 5.5 ± 0.3 s.

Characterization of Solvatochromic Dye 2 (SD2)

The structure of the dye, SD2, is provided in Figure S1; Figure S2 shows the H^1-NMR spectrum for the dye. Synthetic details can be found in the main manuscript.

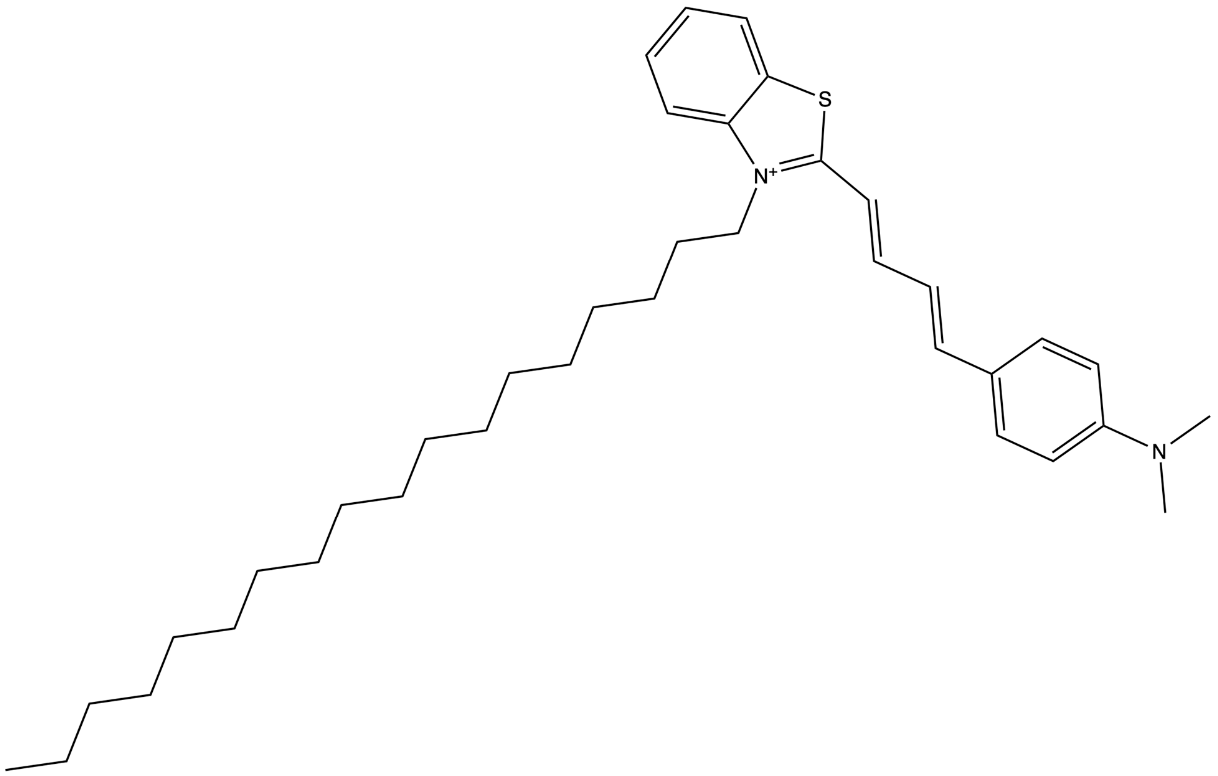

Figure S6. Chemical structure of the dye, SD2. Note that the counter anion, iodide, is not drawn here.

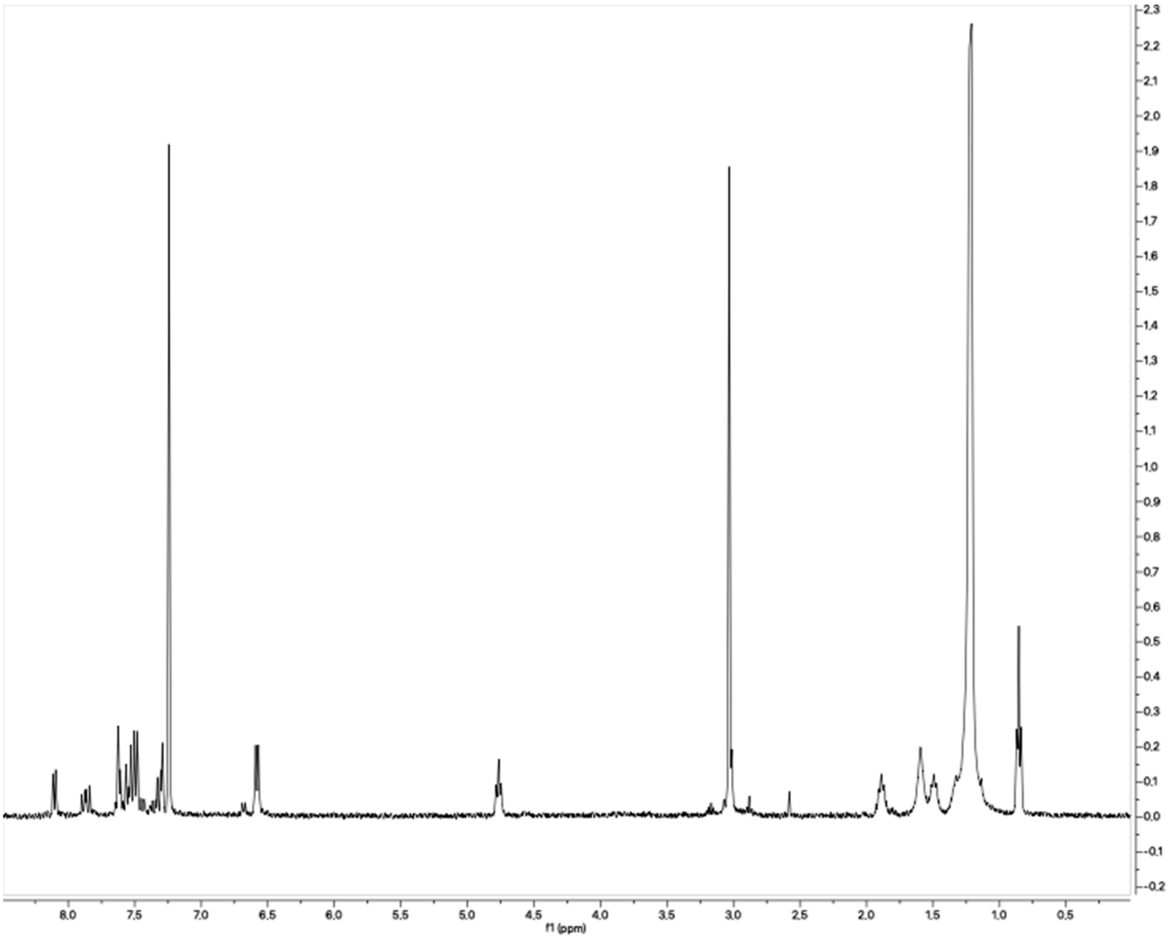

Figure S7. An H^1-NMR spectrum for the synthesized dye, SD2. The NMR spectrum was collected in deuterated chloroform.

Method Comparison

Table S1. Comparison of the advantages and disadvantages of prominent techniques for *in vivo* sodium sensing.

Method	Advantages	Disadvantages	Reference(s)
Na^{23} NMR	• Non-Invasive	• Low spatial resolution<br>• Low signal-to-noise<br>• High equipment costs<br>• Inaccessible to patients with metal implants	[3], [24], [25], [51]
Ion Selective Electrodes (ISEs)	• High signal-to-noise<br>• High sensitivity	• Invasive<br>• Limited spatial resolution requiring multielectrode arrays	[38], [52], [53]
Ionophore-based Optical Sensors (IBOS) Nanosensors	• Minimally invasive<br>• High spatial resolution<br>• High signal-to-noise<br>• High sensitivity<br>• Potential for time-resolved monitoring	• Stability concerns<br>• Toxicity concerns	[38]
Dual-Energy CT	• Non-invasive<br>• Potential for time-resolved monitoring	• Exposure to ionizing radiation<br>• High equipment cost<br>• Long acquisition times	[54]

Table S2. Comparison of SDNaNP to a selection of other prominent sodium nano-sensors reported in the literature. Sensitivity is provided in terms of the detection limit and the dynamic range. Selectivity is given by the selectivity coefficients, a comparison to the potassium concentration required for an equivalent response as to sodium. Fluorescence wavelengths represent the emission wavelengths.

**Sensor**	**Na^+ Sensitivity**	**Selectivity**	**Use of pH-sensitive Dye**	**Toxicity**	**Readout Mode(s)**	**Other**	**Reference**
SDNaNP	Detection Limit: 200 µM	K^+: -3.2	No	Biocompatible	Photoacoustic<br><br>Absorbance (520 nm & 630 nm)	Diameter: 45 nm<br><br>Response time: ~3 s	
PEG-coated Polymeric NS	Resolution of 370 µM<br><br>Half-maximal response of ~20 mM	K^+: Selective at and up to 1 M K^+	Yes	Biocompatible by MTT assay	Fluorescence (λ_{em} = 680 nm)	Response time: order of µs<br><br>Diameter: 123 ± 44 nm	[39],[55]
PCL-based	Detection Limit: 4.6 mM<br><br>Dynamic range centered at 141 mM NaCl	No interference at 15 mM K^+	Yes	Biocompatible/<br><br>Biodegradable	Fluorescence (λ_{em} = 570 nm & 680 nm)	Response time: 48 s<br><br>Diameter: 260 ± 2.2 nm	[40]
Polymer-free Optode	Linear Range: centered at 20 mM<br><br>Ratiometric range that changes 70% from 10 – 1000 mM	K+: Selective by ~1.4 orders of magnitude	Yes	Not reported	Fluorescence (λ_{em} = 580 nm & 680 nm)	Diameter: 254 nm	[41]
Ox-based Nanosensor	Linear Range: ~ 10 – 1000 mM	K^+: Selective by ~1 order magnitude difference	Yes	Not reported	Absorbance (320 nm & 571 nm)<br><br>Fluorescence (λ_{em} = 610 nm)		[42]

Blueberry-CDot Nanosensor	Dynamic Range: 1 – 2000 mM<br><br>$\alpha_{0.5}$ = 200 mM	K^+: 0.4<br><br>Li^+: 0.9	Yes	Not reported	Fluorescence (λ_{em} = 480 nm)		[43]
Glow Sensor	Linear Range: 2.4 – 414 mM<br><br>$\alpha_{0.5}$ = 52 mM	K^+: -2.2<br>Li^+: -3.3	Yes	Not reported	Fluorescence (λ_{em} = 525 nm)	Bulk Optode<br><br>Response time: t_{95} = 9.6 min	[44]
Calix[4]crown-ETH 2439	Detection Limit: 2 µM<br><br>Dynamic Range: ~2-3 orders of magnitude	K^+: < -4.0<br>Ca^{2+}: < -2.4<br>Mg^{2+}: < -3.9	Yes	Not reported	Fluorescence (λ_{em} = ~610 nm & ~700 nm)	Response time: t_{90} = ~10 s	[45]
Biginelli ONP	Detection Limit: 22 nM<br><br>Linear Range: 22 nM – 25 µM	Negligible sensitivity to 40 µM of common non-sodium cations	No	Not reported	Absorbance (330 nm and 380 nm)<br><br>Fluorescence (λ_{em} = 350 nm)	Response time: < 1 minute	[46]
PAMAM Dendrimer-based Nanoprobe (PAMAM-PEG-CoroNa Green)	Linear range: 0 mM - >50 mM<br><br>K_d = 81.2 mM	K+: No interference at 200 mM K+<br><br>Ca^{2+}: No interference at >800 mM Ca^{2+}	No	Biocompatible (*in situ* calibrations performed)	Fluorescence (λ_{em} = ~530 nm)	Diameter: 6.57 ± 0.04 nm<br><br>Response time appropriate for measuring changes due to action potentials	[47]
